# Determinants of orphan drugs prices in France: a regression analysis

**DOI:** 10.1186/s13023-016-0561-5

**Published:** 2017-04-21

**Authors:** Daria Korchagina, Aurelie Millier, Anne-Lise Vataire, Samuel Aballea, Bruno Falissard, Mondher Toumi

**Affiliations:** 1 Mental Health and Public Health, Inserm U669, Maison de Solenn, 97 Boulevard de Port Royal, 75679 Paris, Cedex 14 France; 2 Health Economics and Outcomes Research, Creativ-Ceutical, 215 rue du Faubourg Saint Honoré, 75008 Paris, France; 3 Public Health Department, Research Unit EA 3279, Aix-Marseille University, 27 Bd Jean Moulin, 13385 Marseille, France

**Keywords:** Orphan drugs, Rare disease, Technology assessment, Pricing, Drug costs

## Abstract

**Background:**

The introduction of the orphan drug legislation led to the increase in the number of available orphan drugs, but the access to them is often limited due to the high price. Social preferences regarding funding orphan drugs as well as the criteria taken into consideration while setting the price remain unclear. The study aimed at identifying the determinant of orphan drug prices in France using a regression analysis.

**Methods:**

All drugs with a valid orphan designation at the moment of launch for which the price was available in France were included in the analysis. The selection of covariates was based on a literature review and included drug characteristics (Anatomical Therapeutic Chemical (ATC) class, treatment line, age of target population), diseases characteristics (severity, prevalence, availability of alternative therapeutic options), health technology assessment (HTA) details (actual benefit (AB) and improvement in actual benefit (IAB) scores, delay between the HTA and commercialisation), and study characteristics (type of study, comparator, type of endpoint). The main data sources were European public assessment reports, HTA reports, summaries of opinion on orphan designation of the European Medicines Agency, and the French insurance database of drugs and tariffs. A generalized regression model was developed to test the association between the annual treatment cost and selected covariates.

**Results:**

A total of 68 drugs were included. The mean annual treatment cost was €96,518. In the univariate analysis, the ATC class (*p* = 0.01), availability of alternative treatment options (*p* = 0.02) and the prevalence (*p* = 0.02) showed a significant correlation with the annual cost. The multivariate analysis demonstrated significant association between the annual cost and availability of alternative treatment options, ATC class, IAB score, type of comparator in the pivotal clinical trial, as well as commercialisation date and delay between the HTA and commercialisation.

**Conclusion:**

The orphan drug pricing is a multivariate phenomenon. The complex association between drug prices and the studied attributes and shows that payers integrate multiple variables in decision making when setting orphan drug prices. The interpretation of the study results is limited by the small sample size and the complex data structure.

**Electronic supplementary material:**

The online version of this article (doi:10.1186/s13023-016-0561-5) contains supplementary material, which is available to authorized users.

## Background

For a very long time, rare diseases have been disregarded by the pharmaceutical industry. Small number of potential patients and, thus, low profitability of drugs for rare conditions made them unattractive for investments. In order to guarantee access to appropriate treatments for patients suffering from rare diseases, governments of several countries introduced a special orphan legislation [1]. Granting an orphan designation allows a drug benefiting from a number of incentives, which were developed to boost the return on investments on research and development of orphan designated medicines. The incentives generally include an accelerated and simplified procedure for obtaining a marketing authorization (MA), reduction or fees waiver for the submission, free scientific advice, and market exclusivity [1].

Introducing the orphan legislation led to the fast growth of the number of authorized orphan products. More than 100 orphan drugs have been granted a MA by the European Medicines Agency (EMA) since the establishment of orphan drug designation in 2000 [2]. As of early 2016, there exist more than 1300 valid orphan designations in Europe. Even if the prevalence of a given rare disease is very low, all together orphan drugs represent a significant burden on healthcare budgets, rising concerns about the affordability of orphan drugs [3–6]. Despite the small number of potential patients, some orphan drugs achieved particularly high volumes of sales and became blockbusters. Orphan drug market remains very heterogeneous in term of drugs revenue with a very small number of molecules representing the major part of the market [7]. Moreover, most of authorised products target specific therapeutic areas with high potential profitability, such as oncology or metabolic diseases [8]. Some authors voiced concerns regarding highly lucrative opportunity for manufacturers which orphan drugs represent. The excessive stratification of therapeutic indications and extension of indication to a common or another rare indication lead to significant increase in drug profitability [4].

The question of the social preferences regarding the financing of orphan drugs is unclear. Several studies aimed at analysing the public choice for the allocation of healthcare budget to treat rare diseases [9–11], but showed contradictory results [12]. Furthermore, the appropriateness of standard methods of the health technology assessment (HTA) to appraise value of orphan drugs was frequently questioned in the literature [13, 14]. Given their very high cost, orphan drugs generally do not meet the cost-effectiveness criteria leading to a higher rejection rate for orphan drugs when cost-effectiveness methods are used to decide on reimbursement [15]. Therefore, many authors find imperative to integrate in the evaluation process other criteria, such as disease severity, duration and prognosis of the condition and the absence of alternative treatment option [16–23].

The HTA process in France does not suppose using a cost-effectiveness threshold, but mostly relies on the analysis of clinical aspects. The absence of clearly established criteria to set the price of orphan drugs, leads to the lack of transparency regarding the price. The French system uses two scores to assess drug’s value: actual benefit (AB) and improvement in actual benefit (IAB). AB is based on several criteria such as severity of the disease, its impact on the public health, availability of alternative treatment options, etc. [24]. Insufficient AB leads to the rejection for reimbursement. IAB is the primary driver for the price and reflects drug efficacy as compared to existing treatments [24]. IAB has five levels ranged from I for the major improvement to V for the absence of clinical improvement. IAB from I to III leads to a premium price. More recently health economics assessment has become mandatory for all products that obtain an IAB of III or better and that meet a targeted yearly turnover over twenty million € [25]. However, it is unclear how the IAB is translated to the actual price and which additional parameters are taken into account and how. The price is set by the negotiation with the manufacturer taking into account all submitted evidence. Commitments on sale volumes are negotiated together with the price.

A well-structured, transparent HTA process which integrates all aspects of drug value becomes imperative when the decision on drug funding is associated with considerable ethical and equity concerns, as in case of rare diseases. On the one hand, high cost of orphan drugs requires a significant budget per patient to be allocated to the treatments bringing benefit to a very limited population. On the other, rare diseases are generally associated with a high impact on the morbidity and mortality, and the absence of alternative treatment options [26]. It is crucial to understand decisions of payers on the funding orphan drugs and wherever it reflects the preferences of the society. This study aimed to identify which criteria related to the drug or the disease itself may impact orphan drug prices using France as an illustrative case.

## Methods

All designated orphan drugs holding a MA were identified using the European Medicines Agency’s database [2] of orphan drug designations, regardless wherever the designation has expired or been withdrawn. The drug was included in the final selection if it underwent the HTA in France, when its orphan designation was valid and its price available. The study focused on the price at the moment of drug commercialisation, thus, only first indications were taken into account. In case of granting a MA for several indications simultaneously, they were considered as different drugs and analysed separately. All extractions were conducted in July 2015.

A targeted literature search using Medline base was conducted to identify parameters that may have an impact on orphan drug prices. The search strategy can be found in Additional file 1. The parameters included in the final selection were divided in four categories: drug characteristics (ATC class, treatment line, age of target population), diseases characteristics (severity, prevalence, availability of alternative therapeutic options), HTA details (AB and IAB scores, delay between the HTA and commercialisation), and study characteristics (type of study, comparator, type of endpoint). The details on the structure of the parameters, as well as sources of data are presented in Table 1.

**Table 1:** Characteristics of orphan drugs included in the analysis

Variable	Values	Source
Total number	68	
Disease characteristics		
Prevalence per 10,000, mean (sd)	1.19 (1.08)	Public summary of opinion on orphan designation
Severity, n(%)	Severe 13 (19%); Not severe 55 (81%)	Public summary of opinion on orphan designation
Availability of alternative treatments, n(%)	None 22 (32%); One 10 (15%); Several 30 (44%); Non-pharmacological 6 (9%)	
Drug characteristics		
ATC class, n(%)	A 14 (21%); B 3 (4%); C 4 (6%); G 1 (1%); H 3 (4%); J 3 (4%); L 32 (47%); N 5 (7%); R 1 (1%); V 2 (3%)	European public assessment report
Treatment line, n(%)	First 40 (59%); Subsequent 28 (41%)	HTA report
Age of targeted population, n(%)	Adults 34 (50%); Paediatric 15 (22%); Adults and paediatric 14 (21%); Elderly 5 (7%)	European public assessment report
HTA details		
AB score, n(%)	Substantial 64 (94%); Moderate 2 (3%); Low 1 (1%); NR 1 (1%)	HTA report
IAB score, n(%)	I 6 (9%); II 25 (37%); III 14 (21%); IV 15 (22%); V 6 (9%); NR 2 (3%)	HTA report
Commercialisation date	See Fig. 2	Ameli database
Delay between HTA and commercialisation, mean (sd) months	6.68 (10.44)	HTA report/ Ameli database
Study characteristics		
Type of study, n(%)	Phase II 14 (21%); Phase III 43 (63%); Other 11 (16%)	HTA report
Comparator, n(%)	None 20 (29%); Placebo 27 (40%); Active 18 (26%); NR 3 (4%)	HTA report
Endpoint, n(%)	Surrogate 45 (66%); Hard 12 (18%); NR 11 (16%)	HTA report
Cost		
Annual treatment cost, mean (sd)	€96,518 (€169,942)	Ameli database/European public assessment report

ATC class: *A* Alimentary tract and metabolism; *B* Blood and blood forming organs; *C* Cardiovascular system; *G* Genito-urinary system and sex hormones; *H* Systemic hormonal preparations, excluding sex hormones and insulins; *J* Antiinfectives for systemic use; *L* Antineoplastic and immunomodulating agents; *N* Nervous system; *R* Respiratory system; *V* Various

Disease prevalence and severity were available from the public summaries of opinion on orphan designation of the EMA. A disease was classified as severe if it was described as ‘serious life-threating’ condition in the summary on orphan designation. If the age of target population was not clearly stated in the European public assessment reports (EPAR), it was determined based on the mean patient’s age in the pivotal clinical trial.

The details of the HTA were extracted from the reports of the French HTA agency which are publicly published on its official website [27]. HTA reports also provided information on the submitted evidence, treatment line and alternative therapeutic options available in France.

The annual treatment cost for each drug was calculated based on dosing and ex-factory drug prices at launch. The treatment regimen was extracted from the Summary of product characteristics (SmPC) published in EPARs. In case the drug was not intended for a long term therapy or in case life expectancy was lower than one year, the treatment duration was set at the same duration as in the pivotal clinical trial. An average weight of 70 kg and body surface of 1.75 m^2^ was used to calculate the daily dose for adult patients if any adjustment was needed. In case of drugs indicated for paediatric patients, weight and body surface used for the calculations corresponded to the mean age of patients in the pivotal clinical trial [28]. When the recommended daily dose was given by an interval, the mean value was used. In case of dose adjustment based on laboratory parameters, the daily dose used to calculate the treatment cost was set at the same value as in the pivotal clinical trial.

Listed drug prices in France are publicly published in the insurance database of drugs and tariffs (Ameli database) [29]. Price and reimbursement history is available for all drugs except the drugs for hospital use only. However, prices are also available for exceptionally expensive hospital only medicines that are included in a special funding program or for drugs distributed in retail through hospital pharmacies. The date of the first publication on the reimbursement of the drug was considered to be the date of commercialisation.

Descriptive statistics were conducted for each available variable. Then, the correlation structure between the annual treatment costs and the covariates, as well as between the covariates themselves, was studied in a univariate analysis. Chi-2 test or Fisher’s exact test were considered for categorical variables and Student’s or Welch’s t-test were considered for continuous variables. Pearson correlation and Spearmen correlation coefficients were calculated in case both variables were continuous.

Then, a generalized regression model using several distributions was developed to identify price determinants. The choice of covariates to be included was based on the results of the univariate analysis and expert opinion. All covariates that showed an association with the annual cost under *p* ≤ 0.20 threshold were to be included in the model. Additionally, two experts were consulted to identify covariates that should theoretically have a correlation with drug cost which might have not been captured in the univariate analysis. The consulted experts considered a number of parameters such as disease characteristics, IAB score, ATC class and treatment line to be of particular interest and suggested their inclusion in the regression model regardless whether they have shown a significant association with annual treatment cost in the univariate analysis. Additionally, it was decided to test a regression model containing all remaining covariates. The choice of the model in term of included covariates and distribution was based on the root-mean-square error (RMSE) which was calculated as a standard deviation of the differences between predicted values and observed values. A model with the smallest associated RMSE was retained. All analyses were conducted in SAS 9.3.

## Results

**Fig. 1:**
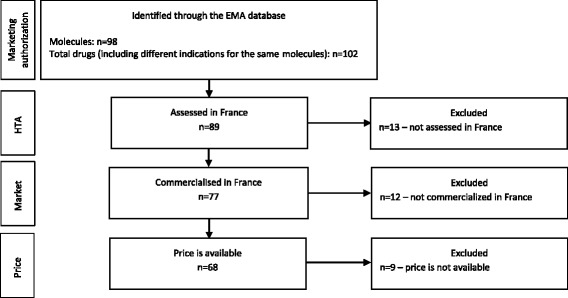
Selection of orphan drugs to be included in the analysis

Almost 100 orphan molecules holding a MA were identified through the EMA database of orphan drug designations. Four of them were approved for two indications, giving in total 102 drugs to be analysed. Of these 102 drugs, 89 have undergone the HTA in France and 77 were on the market at the moment of the analysis. For 68 of them the price was available (Fig. 1).

**Fig. 2:**
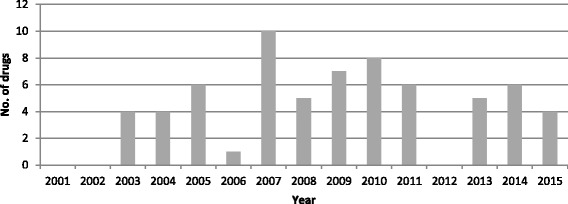
Number of analysed orphan drugs per commercialisation year

The characteristics of the selected drugs are summarised in Table 1. Most of the drugs were from ATC class A (alimentary tract and metabolism, 21%) and L (antineoplastic and immunomodulating agents, 47%). Almost 20% of the diseases were described as severe in the public summary of opinion on orphan designation. For most of the diseases, there existed several alternative treatment options. The prevalence ranged from 0.001 to 5 patients per 10,000 habitants with mean of 1.19 per 10,000.

Despite the small size of the target population, 63% of drugs were studied in phase III clinical trials and 21% in phase II. However, 30% of them were non comparative and the majority presented results based on a surrogate endpoint (66%). Alternative study designs were mainly retrospective studies, cohorts and case reports. Almost all the drugs (94%) were classified as bringing a substantial benefit to patients (AB score). The IAB score from I to III which guaranteed a premium price was granted to 67% of the drugs. At this stage, it was decided to exclude AB score from the further analysis since this parameter did not allow differentiating between the drugs seeing that only tree drugs obtained the score less than ‘substantial’. The distribution of the number of commercialized orphan drugs by year is presented in Fig. 2.

**Fig. 3:**
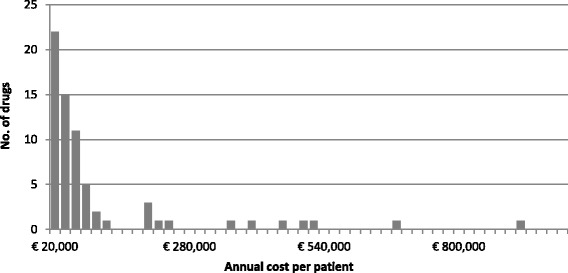
Histogram of annual treatment cost of orphan drugs in France

The annual treatment costs ranged from €1474 to €912,000 per patient with the mean and the median of €96,518 and €35,644, respectively. Among the analysed molecules, 12 drugs had a considerably higher treatment cost compared to other drugs (Fig. 3).

In the univariate analysis, disease prevalence showed an inverse statistically significant correlation with annual treatment cost (*p* = 0.02); also ATC class (*p* = 0.01) and availability of alternative treatment options (*p* = 0.02) were significantly association with annual cost (see Table 2). Additionally, the *p* value for IAB score (*p* = 0.20) and treatment line (*p* = 0.13) were below 0.20 threshold. All the identified covariates were initially present in the selection to be tested regardless the results of the univariate analysis.

**Table 2:** Results of the univariate analysis: association between the annual treatment costs and the covariates

Categorical variables					
	Mean	Median	95% CI	Min-Max	*p*-value
Severity					
Severe	96,385 (174,479)	25,069	[−9052; 201,821]	[1474; 509,600]	*p* = 1.00
Not severe	96,549 (170,493)	38,482	[50,459 ; 142,640]	[1524; 912,600]	
Availability of alternative treatments					
None	131,253 (211,910)	53,746	[37,297; 225,209]	[2080; 912,600]	*p* = 0.02
One	70,280 (71,467)	40,746	[19,155; 121,404]	[1474; 198,640]	
Several	45,572 (92,292)	18,695	[11,109; 80,034]	[1500; 481,581]	
Non-pharmacological	267,615 (287,063)	205,962	[−33,638; 568,869]	[3163; 676,260]	
ATC class					
L	44,473 (60,908)	29,525	[22,513; 66,432]	[1474; 347,100]	*p* = 0.01
C	36,044 (8004)	37,108	[23,309; 48,780]	[23,309; 48,780]	
A	280,878 (277,242)	197,886	[120,803; 440,953]	[1524; 912,600]	
J	11,278 (4622)	13,236	[−203; 22,760]	[6000; 14,600]	
N	10,682 (10,669)	7688	[−2565; 23,930]	[2637; 29,200]	
H	186,122 (237,122)	52,560	[−402,923; 775,166]	[45,905; 459,900]	
B	45,349 (15,033)	37,799	[8006; 82,692]	[35,588; 62,660]	
V, R, G	70,488 (110,625)	22,614	[−105,542; 246,517]	[2080; 234,642]	
Treatment line,					
First	140,828 (210,443)	44,142	[73,525; 208,131]	[1474; 912,600]	*p* = 0.13
Subsequent	33,218 (25,149)	25,655	[23,466; 42,969]	[2637; 95,265]	
Age of targeted population					
Adults	44,554 (46,449)	34,219	[28,347; 60,761]	[1500; 198,640]	*p* = 0.53
Paediatric	262,492 (292,292)	192,477	[100,626; 424,359]	[1474; 912,600]	
Adults and paediatric	66,384 (100,464)	33,566	[8378; 124,390]	[1524; 347,100]	
Elderly	36,321 (12,527)	44,604	[20,767; 51,875]	[20,440; 46,888]	
MA date					
2202–2003	122,186 (166,320)	33,941	[−16,860; 261,233]	[4623; 481,581]	*p* = 0.66
2004–2005	102,040 (182,695)	37,800	[−66,924; 271,005]	[1500; 509,600]	
2006–2007	160,681 (276,804)	38,482	[7393; 313,971]	[1524; 912,600]	
2008–2009	65,466 (111,874)	43,680	[3512; 127,420]	[5451; 459,900]	
2010–2011	63,544 (80,581)	32,394	[−21,021; 148,108]	[2637; 219,889]	
2012–2013	88,466 (116,624)	46,888	[10,117; 166,815]	[3163; 382,200]	
2014–2015	20,806 (16,447)	20,775	[3546; 38,066]	[1474; 41,366]	
IAB score					
I	101,666 (186,914)	33,941	[−94,488; 297,820]	[4745; 481,581]	*p* = 0.20
II	148,647 (206,028)	62,660	[63,603; 233,691]	[12,093; 912,600]	
III	111,831 (199,604)	49,724	[−3417; 227,080]	[2080; 676,260]	
IV	16,724 (16,359)	9265	[7664; 25,783]	[1474; 45,905]	
V	50,807 (83,243)	19,758	[−36,551; 138,165]	[6000; 219,889]	
Type of study					
Phase II	48,782 (49,992)	40,038	[19,917; 77,646]	[1500; 198,640]	*p* = 0.40
Phase III	116,757 (197,406)	38,483	[56,004; 177,510]	[2080; 912,600]	
Other	78,156 (145,794)	14,600	[−19,790; 176,102]	[1474; 481,581]	
Comparator					
No	90,783 (153,970)	33,566	[18,723; 162,844]	[1500; 481,581]	*p* = 0.50
Placebo	128,831 (226,137)	35,588	[39,374; 218,288]	[2637; 912,600]	
Active	68,347 (73,068)	44,142	[32,012; 104,683]	[32,012; 104,683]	
Endpoint					
Surrogate	110,012 (187,794)	41,366	[53,593; 166,431]	[1500; 912,600]	*p* = 0.50
Hard	71,632 (113,019)	32,190	[−177; 143,441]	[2080; 382,200]	
Continuous variables					
	Pearson Correlation	*p*-value	Spearman Correlation	*p*-value	
Prevalence	−0.32472	*p* = 0.01	−0.28433	*p* = 0.02	
Delay between HTA and commercialisation	0.04208	*p* = 0.73	−0.19956	*p* = 0.10	

ATC class: *A* Alimentary tract and metabolism; *B* Blood and blood forming organs; *C* Cardiovascular system; *G* Genito-urinary system and sex hormones; *H* Systemic hormonal preparations, excluding sex hormones and insulins; *J* Antiinfectives for systemic use; *L* Antineoplastic and immunomodulating agents; *N* Nervous system; *R* Respiratory system; *V* Various

Costs are given in €

Based on the RMSE criteria better fit was obtained using negative binomial distributions and including all covariates. The results of the regression analysis are presented in Table 3. Availability of alternative treatment options, ATC class, IAB score, type of comparator in the pivotal clinical trial, as well as commercialisation date and delay between the HTA and commercialisation were significantly associated with annual treatment cost. Higher treatment costs were observed for the diseases where no alternative treatments (*p* = 0.0003) or only non-pharmacological therapies were available (*p* < 0.0001). Regarding the ATC class, A (alimentary tract and metabolism, *p* < 0.0001), C (cardiovascular system, *p* = 0.0254) and H (systemic hormonal preparations, excluding sex hormones and insulins, *p* < 0.0001) were associated with significantly higher treatment costs as compared with L (antineoplastic and immunomodulating agents), while drugs from J (anti-infective for systemic use, *p* = 0.0002) class and the combined category corresponding to V (various), R (respiratory system), G (genito-urinary system and sex hormones) classes (*p* < 0.0001) had lower prices.

**Table 3:** Results of the generalized regression model

Parameter	*p*-value for type 3 statistics	Estimate	SE	Wald 95% Confidence Limits		*p*-value
Intercept		6.7811	0.8734	5.0693	8.4929	<.0001
Prevalence	0.1073	0.1632	0.1016	−0.0360	0.3624	0.1084
Age						
Adults	0.2753	−0.1691	0.3603	−0.8754	0.5371	0.6388
Adults, paediatric		0.0268	0.4307	−0.8173	0.8709	0.9503
Elderly		−0.8188	0.5580	−1.9124	0.2749	0.1423
Paediatric		0.0000	0.0000	0.0000	0.0000	.
Alternative						
No	0.0006	1.1042	0.3062	0.5041	1.7043	0.0003
Non-pharmacological		1.7580	0.4483	0.8793	2.6368	<.0001
One		0.1077	0.3216	−0.5227	0.7381	0.7377
Several		0.0000	0.0000	0.0000	0.0000	.
Severity						
Not Severe	0.2602	0.2505	0.2196	−0.1799	0.6809	0.2540
Severe		0.0000	0.0000	0.0000	0.0000	.
ATC						
A	<.0001	2.4019	0.4458	1.5282	3.2755	<.0001
B		0.6939	0.4266	−0.1422	1.5300	0.1038
C		0.9708	0.4344	0.1194	1.8223	0.0254
H		2.1675	0.4308	1.3232	3.0118	<.0001
J		−2.6030	0.7054	−3.9855	−1.2205	0.0002
N		0.6858	0.5076	−0.3091	1.6807	0.1767
V, R, G		−1.6830	0.3856	−2.4387	−0.9273	<.0001
L		0.0000	0.0000	0.0000	0.0000	.
Line						
First	0.4234	0.2195	0.2738	−0.3171	0.7561	0.4227
Subsequent		0.0000	0.0000	0.0000	0.0000	.
IAB						
I	<.0001	1.2641	0.4322	0.4169	2.1113	0.0035
II		1.2281	0.3514	0.5394	1.9168	0.0005
III		−0.1662	0.3763	−0.9037	0.5714	0.6588
IV		−0.5557	0.3711	−1.2830	0.1716	0.1343
V		0.0000	0.0000	0.0000	0.0000	.
Study						
Other	0.6266	0.6525	0.7739	−0.8643	2.1693	0.3992
Phase II		0.1203	0.3037	−0.4748	0.7155	0.6919
Phase III		0.0000	0.0000	0.0000	0.0000	.
Comparator						
Active	<.0001	1.5872	0.3192	0.9616	2.2128	<.0001
No		0.4590	0.3231	−0.1743	1.0923	0.1555
Placebo		0.0000	0.0000	0.0000	0.0000	.
Endpoint						
Hard	0.1481	−0.4280	0.2905	−0.9973	0.1413	0.1406
Surrogate		0.0000	0.0000	0.0000	0.0000	.
Commercialisation date						
2002–2003	0.0071	−0.2061	0.5230	−1.2311	0.8188	0.6934
2004–2005		−0.8557	0.5956	−2.0232	0.3117	0.1508
2006–2007		0.2278	0.4836	−0.7201	1.1756	0.6377
2008–2009		0.0529	0.4049	−0.7407	0.8466	0.8960
2010–2011		−0.2592	0.5049	−1.2487	0.7304	0.6077
2012–2013		0.9553	0.4206	0.1310	1.7797	0.0231
2014–2015		0.0000	0.0000	0.0000	0.0000	.
Delay HTA/Commercialisation	0.0048	−0.0318	0.0107	−0.0527	−0.0108	0.0029
Dispersion		0.2105	0.0385	0.1471	0.3011	

ATC class: *A* Alimentary tract and metabolism; *B* Blood and blood forming organs; *C* Cardiovascular system; *G* Genito-urinary system and sex hormones; *H* Systemic hormonal preparations, excluding sex hormones and insulins; *J* Antiinfectives for systemic use; *L* Antineoplastic and immunomodulating agents; *N* Nervous system; *R* Respiratory system; *V* Various

Comparison versus an active comparator in the pivotal clinical trial also showed a significant positive impact on the annual cost (*p* < 0.0001). An inverse relationship was observed between the cost and the delay between the HTA and commercialisation (*p* = 0.0029) with higher costs associated with shorter delay. Significantly higher costs were also observed for IAB I (*p* = 0.0035) and II (*p* = 0.0005).

## Discussion

Many authors suggested using multiple criteria to inform the decision on orphan drug prices and proposed a list of attributes to be considered in the decision making. The most frequently cited ones include availability of alternative treatments [16, 18, 30], disease severity and prognosis with currently available treatment [16, 18, 19, 22, 30, 31], size of target population [16, 22, 30], clinical effectiveness and its magnitude [16, 18, 19, 22, 30, 31], safety profile [18, 30], robustness of submitted evidence [18, 19, 30, 31], social impact [18, 31], innovation profile and cost of development [16, 18], budget impact [22, 31] and some others. In our study we analysed wherever a number of these parameters were associated with orphan drugs prices in France. The choice of parameters to be included was based on the availability of data. For instance, many authors suggested the cost of undertaken research to be considered in the evaluation process. However, manufacturers rarely make the information on the cost of drug development publicly available. A significant correlation with annual treatment cost was observed for availability of alternative treatment options, ATC class, IAB score, type of comparator in the pivotal clinical trial, as well as for commercialisation date and delay between the HTA and commercialisation.

The analysis showed that the rarity itself seems not to be valued by payers. In the univariate analysis a statistically significant correlation was observed between the disease prevalence and the annual treatment costs. Higher cost was associated with lower prevalence. However, after adjustment for other parameters this association was no longer significant. Another result of the univariate analysis was the absence of correlation between the treatment cost and the IAB score, which is considered to be the primary driver for drug prices in France. However, this association was evidenced in the regression model. The contradiction between the results of the univariate analysis and the regression models is well known in statistics. On the one hand, it may demonstrate the complex association between drug prices and the studied attributes and shows that payers integrate multiple variables in decision making when setting orphan drug prices. This would mean the orphan drug pricing is a multivariate phenomenon. On the other, the absence of significant correlation in the univariate analysis and its presence in the multivariate one, may suggest several methodological issues. A number of scenarios leading to such results have been described in the literature including the effect of unbalanced sample size and a large within group variation, relative to between group variation [32]. Both were the case of the presented study. Moreover, it could suggest the presence of unstudied interactions between the included covariates which were impossible to consider in the analysis due to the small sample size. Nevertheless, these problems were caused by the structure of the data and its availability and are difficult to overcome.

The lack of alternative treatment options represented high unmet needs for many orphan drugs. Conditions with no alternative treatments or only non-pharmacological alternative therapies were associated with higher annual costs. This finding is in line with the most of frameworks that were proposed in the literature for the assessment of orphan drugs and which suggested that the availability of alternative treatment options should be taken into account [16, 18–20, 31].

Higher costs were also associated with shorter delay between the HTA and drug commercialisation. Indeed, in case of a severe disease with high unmet needs or a particular drug efficacy, payers may be willing to ensure a faster access to the therapy and are more likely to accept higher prices. Longer window between HTA and price may suggest complex negotiation and disagreement on the product value.

Surprisingly, there was no significant correlation between the diseases severity and the treatment cost. It should be noted that in this study disease severity was evaluated based on the disease description in the public summaries of opinion on orphan designation available from the official EMA website. It is not clear how the severity was assessed and whether it was reported consistently. However, no reliable systematic classification on the severity of rare disease exists [33]. Disease severity is one of the mandatory criteria for granting an orphan designation. High severity level of rare diseases that were considered in the study was also acknowledged through assigning an ‘important’ AB score to the most of orphan drugs. Therefore, a very fine severity classification is needed to allow differentiating between treatments.

The only attribute related to study characteristics that demonstrated a significant association with the treatment cost was the type of comparator. Higher treatment costs were observed when the clinical trial was conducted versus an active comparator. On the one hand, the use of an active comparator means that an alternative treatment option exist, on the other, it allows a more robust estimation of the added value.

Some previous studies demonstrated the association between the disease prevalence and the annual treatment costs for orphan drugs [5, 34, 35]. However, the tentative to describe orphan drug prices based on predefined disease and drug characteristics using a robust methodology remains very limited. Aballéa et al. [36] studied orphan drug prices in five European countries: France, Germany, Italy, Spain and United Kingdom. The developed Poisson model did not reveal any statistically significant correlation between the annual treatment cost and the studied parameters that included the prevalence, prognosis, age of target population, seriousness of the condition, number of alternative treatments, course of illness, as well as year of approval, trial size, number of trials, ATC code, and evidence of benefit. Low disease prevalence and low number available therapeutic alternatives seemed, however, be associated with increased yearly prices. A noteworthy detail is that the analysis included only 19 orphan drugs and was likely to be underpowered to capture associations.

More recently, Pivacet et al. [33] conducted a regression analysis in order to identify the determinants of orphan drug prices in Belgium, the Netherlands, Czech Republic, France, Italy and the United Kingdom. The list of tested explanatory variables included several disease and drug characteristics such as availability of alternative treatment options, impact on the overall survival and patient’s quality of life, drug formulation, availability of evidence from randomized controlled trials, whether the drug was repurposed, indicated to treat oncology diseases or whether it had multiple indications, and some other. Disease prevalence was integrated as a binary variable which allowed differentiation between rare and ultra-rare diseases. The study uses multiple linear regression analysis with stepwise removing variables with significance levels above the 0.05. The results demonstrated that repurposed orphan drugs, oral formulation and availability of alternative treatments were associated with lower annual treatment costs, while multiple orphan indications, drugs for chronic treatments and improvement in overall survival or QoL were associated with higher annual treatment costs. In our study we did not use repurposing as a variable. However, the philosophy of the French pricing committee (CEPS) is to reward the risk while repurposing is considered as a low risk strategy especially when supported by off label use [25].

Grand et al. [37] used a linear regression model to study the association between the daily cost of orphan drugs in France and a number of parameters including IAB score, number of indications, disease prevalence, inclusion in the list for a special funding program and distribution in retail through hospital pharmacies. Significantly lower costs were observed for molecules with only one indication, IAB score V and associated with higher prevalence. It may be, however, questioned whether the use of daily costs as dependant variable is relevant, as the life expectancy (which is often less than one year) and treatment regimen vary a lot from one condition to another.

To our knowledge, our study is the only one which was interested in orphan drug prices at launch and took into account exclusively first approved indications. The main reason for doing so was that drugs are generally offered a weighted average price when multiple indication are granted and new revision of price include the increase size of the target population. Considering several indications may create confusion. From this point of view, including the number of indications in the analysis does not seem to be relevant, as this information was obviously not available for payers at the moment of price negotiation. From the identified 98 orphan molecules, only 4 were initially approved for more than one indication. Separate indications for the same drugs were considered as independent drugs in our analysis.

The study has some limitations. First of all, the number of commercialised orphan drugs with published prices remains relatively small, however this study considered more drugs than previously published ones. The analysis was based on 68 drugs, at the same time some covariates had an important number of level.

Secondly, the prices for hospital only drugs are not available in France. The prices for this type of medicines are set by the negotiation with each hospital and not publicly published. However, they may be available for drugs that are distributed in retail through hospital pharmacies or for very costly drugs that are distributed through a special funding program. The maximum price of such drugs is available and may be considered as a reasonable price proxy. In total from 77 orphan drugs commercialised in France, the price was not available for nine molecules. This number remains limited and is unlikely to significantly affect the results.

Third, orphan drug prices may be impacted by some other factors that were not identified in our analysis or that could not be quantified. More specifically, many of proposed frameworks to set the price of orphan drugs include the therapeutic effect or the level of innovation as parameters [16, 18–20, 22, 31]. However, in our study we assumed that the IAB score integrated this information since it represents the incremented therapeutic value of the drug compared to existing treatment options. Other suggested criteria were the cost or amount of undertaken research [16, 21, 23]. These data are generally confidential and the price is not set based on a cost plus model but on value generated by new therapy. We believe we capture the therapy additional value through IAB. No cost-effectiveness data was considered in the analysis either. The main reason is that the economic evaluation was not mandatory in France before 2013. Since 2013 cost-effectiveness studies has been required for all drugs with IAB score from I to III, if their yearly budget impact is above 20 million €. In practice this is not the case for many drugs. The French CEPS assume a variable ICER threshold and accept very high ICER for orphan drugs. Finally, the economic evaluation in France is not supposed to affect the listed price but the confidential rebate. Despite these changes in the HTA procedure, the assessment is still mainly based on the clinical benefit.

Finally, actual drug prices may be different from listed prices since France applies volume agreements as the main cost-containment measure. The details of the agreements are confidential and are not accessible for such research, making it impossible to estimate actual orphan drug prices. The presented study considered listed orphan drug prices to calculate annual treatment cost.

## Conclusion

The study analysed the association between the annual treatment cost of orphan drugs in France and a number of disease and drug characteristics. A significant association was observed for the availability of alternative treatment options, ATC class, IAB score, type of comparator in the pivotal clinical trial, as well as for commercialisation date and delay between the HTA and commercialisation. The study demonstrated a complex association between the annual cost and the covariates which cannot be explained only by the IAB score.

Decisions on orphan drug prices remain non transparent in most of cases. A robust comprehensive framework is needed to assess orphan drugs value. Several methodologies have been proposed mostly based on the multi-criteria decision analysis [16, 18, 19, 22, 30, 31]. The current approach complies partially with the proposed criteria to set the price.

## Additional file

Additional file 1: Strategy of the targeted literature search. Strategy of the targeted literature search which was conducted to identify the covariates to be included in the analysis. (DOCX 15 kb)

## References

[1] Gammie T, Lu CY, Babar ZU-D (2015). Access to orphan drugs: a comprehensive review of legislations, regulations and policies in 35 countries. PLoS ONE.

[2] European medicines Agency. Rare disease designations http://www.ema.europa.eu/ema/index.jsp?curl=pages/medicines/landing/orphan_search.jsp&mid=WC0b01ac058001d12b. Accessed 22 Apr 2016.

[3] Roos JC, Hyry HI, Cox TM (2010). Orphan drug pricing may warrant a competition law investigation. BMJ.

[4] Cote A, Keating B (2012). What is wrong with orphan drug policies?. Value Health.

[5] Simoens S (2011). Pricing and reimbursement of orphan drugs: the need for more transparency. Orphanet J Rare Dis.

[6] Simoens S, Cassiman D, Dooms M, Picavet E (2012). Orphan drugs for rare diseases: is it time to revisit their special market access status?. Drugs.

[7] DataMonitor Healthcare. Orphan drug trends 2011. Reference code: HC00143-001; 2011.

[8] Rodrigues J, Korchagina D, Rémuzat C, Brunet J, Tavella F (2014). Orphan drug approvals in Europe: historical review and trends. Value Health.

[9] Excellence NIfHaC (2004). Citizens council reports: ultra orphan drugs.

[10] Linley WG, Hughes DA (2013). Societal views on NICE, cancer drugs fund and value-based pricing criteria for prioritising medicines: a cross-sectional survey of 4118 adults in Great Britain. Health Econ.

[11] Desser AS, Gyrd-Hansen D, Olsen JA, Grepperud S, Kristiansen IS (2010). Societal views on orphan drugs: cross sectional survey of Norwegians aged 40 to 67. BMJ.

[12] Drummond M, Towse A (2014). Orphan drugs policies: a suitable case for treatment. [Review]. Eur J Health Econ.

[13] Clarke JT (2006). Is the current approach to reviewing new drugs condemning the victims of rare diseases to death? A call for a national orphan drug review policy. CMAJ.

[14] Panju AH, Bell CM (2010). Policy alternatives for treatments for rare diseases. Can Med Assoc J.

[15] Adams B. Rare drugs - raw deal? Pharma Times Mag. 2013:35–7.

[16] Hughes-Wilson W, Palma A, Schuurman A, Simoens S (2012). Paying for the Orphan Drug System: break or bend? Is it time for a new evaluation system for payers in Europe to take account of new rare disease treatments?. Orphanet J Rare Dis.

[17] Simoens S, Picavet E, Dooms M, Cassiman D, Morel T (2013). Cost-effectiveness assessment of orphan drugs: a scientific and political conundrum. Appl Health Econ Health Policy.

[18] Sussex J, Rollet P, Garau M, Schmitt C, Kent A, Hutchings A (2013). A pilot study of multicriteria decision analysis for valuing orphan medicines. Value Health.

[19] Commission E (2014). Transparent value framework (Platform on access to medicines in Europe - Working Group on Mechanism of coordinated access to orphan medicinal products).

[20] Paulden M, Stafinski T, Menon D, McCabe C (2015). Value-based reimbursement decisions for orphan drugs: a scoping review and decision framework. Pharmacoeconomics.

[21] Fellows GK, Hollis A (2013). Funding innovation for treatment for rare diseases: adopting a cost-based yardstick approach. Orphanet J Rare Dis.

[22] Winquist E, Bell CM, Clarke JT, Evans G, Martin J, Sabharwal M, Gadhok A, Stevenson H, Coyle D (2012). An evaluation framework for funding drugs for rare diseases. Value Health.

[23] Valverde AM, Reed SD, Schulman KA (2012). Proposed ‘grant-and-access’ program with price caps could stimulate development of drugs for very rare diseases. [Review]. Health Aff.

[24] Rémuzat C, Toumi M, Falissard B (2013). New drug regulations in France: what are the impacts on market access? Part 1 - Overview of new drug regulations in France.

[25] French Healthcare Products Pricing Committee (CEPS). 2014/2015 Annual report. 2015. http://social-sante.gouv.fr/IMG/pdf/rapport_annuel_2014_version_anglaise.pdf. Accessed 15 Aug 2016

[26] Field MJ, Thomas F (2011). Boat ECoARDRaOPDBoHSPIoM: rare diseases and orphan products: accelerating research and development.

[27] Haute Autorité de Santé – HAS. http://www.has-sante.fr/portail/. Accessed 30 Apr 2016

[28] Group WMGRS. WHO Child Growth Standards: Length/height-for-age, weight-for-age, weight-for-length, weight-for-height and body mass index-for-age: Methods and development. Geneva: World Health Organization; 2006. p. 312.

[29] Assurance Maladie. Base des Médicaments et Informations Tarifaires. http://www.codage.ext.cnamts.fr/codif/bdm_it/index.php?p_site=AMELI. Accessed 31 Jul 2015.

[30] Wagner M, Khoury H, Willet J, Rindress D, Goetghebeur M (2016). Can the EVIDEM framework tackle issues raised by evaluating treatments for rare diseases: analysis of issues and policies, and context-specific adaptation. Pharmacoeconomics.

[31] National Institute for Health and Care Excellence (2013). Highly Specialised Technologies programme: Interim process and methods.

[32] Lo SK, Li IT, Tsou TS, See L (1995). Non-significant in univariate but significant in multivariate analysis: a discussion with examples. Changgeng Yi Xue Za Zhi.

[33] Picavet E, Morel T, Cassiman D, Simoens S (2014). Shining a light in the black box of orphan drug pricing. Orphanet J Rare Dis.

[34] Richards T. Orphan diseases: which ones do we adopt? BMJ. 2008;337:a1225. 10.1136/bmj.a1225 18694885

[35] Messori A, Cicchetti A, Patregani L (2010). Orphan drugs. Relating price determination to disease prevalence. BMJ.

[36] Aballea S, Toumi M, Vataire AL, Millier A, Lamure M. PHP6 Quantitative analysis of the influence of disease and product characteristics on orphan drug prices in Europe. ISPOR 15th Annual International Meeting, Atlanta, GA, USA, May 15-19, 2010. http://www.creativ-ceutical.com/sites/default/files/ISPOR%20International_Creativ-Ceutical_Orphan%20Drugs%20Poster.pdf. Accessed 30 Apr 2016

[37] Grand H, Samson AL, Aulois-Griot M (2014). Orphan drug pricing in France: influence of main factors. Value Health.

