# Supplementary material for: Determinants of orphan drugs prices in France: a regression analysis
Source: Orphanet J Rare Dis. 2017 Apr 21;12:75. doi: 10.1186/s13023-016-0561-5 (PMC5399414; doi:10.1186/s13023-016-0561-5)
Supplement: Additional file 1: — Strategy of the targeted literature search. Strategy of the targeted literature search which was conducted to identify the covariates to be included in the analysis. (DOCX 15 kb) [file 13023_2016_561_MOESM1_ESM.docx]

Strategy of the targeted literature search

| 1 | Rare disease/Orphan drugs | exp Orphan Drug Production/ | 883 |
| --- | --- | --- | --- |
| 2 |  | exp Rare Diseases/ | 6800 |
| 3 |  | (rare disease? or orphan disease? or rare disorder? or orphan disorder? or rare condition? or orphan drug? or orphan product? or ultrarare disease? or highly specialized technolog* or orphan medicinal product? or ultraorphan drug? or ultraorphan disease? or neglected disease?).ti,ab. | 36154 |
| 4 | HTA | exp Economics, Pharmaceutical/ or exp Economics, Medical/ or exp Economics/ | 522024 |
| 5 |  | exp Decision Making/de, es [Drug Effects, Ethics] | 13869 |
| 6 |  | exp Technology Assessment, Biomedical/ | 9612 |
| 7 |  | exp Insurance, Health, Reimbursement/ or exp Reimbursement Mechanisms/ or exp Reimbursement, Incentive/ | 40829 |
| 8 |  | exp Evaluation Studies as Topic/ec [Economics] | 1560 |
| 9 |  | exp "Costs and Cost Analysis"/ | 195358 |
| 10 |  | exp Drug Costs/ | 13127 |
| 11 |  | exp Health Policy/ec [Economics] | 8410 |
| 12 |  | exp Health Resources/ec, es [Economics, Ethics] | 2253 |
| 13 |  | exp Social Values/ | 18636 |
| 14 |  | exp Quality-Adjusted Life Years/ | 8146 |
| 15 |  | exp Cost-Benefit Analysis/ | 64846 |
| 16 |  | (multicriteria decision analys#s or multi-criteria decision analys#s or multi criteria decision analys#s or mcda).mp. | 415 |
| 17 |  | (economic evaluation or technology assessment).ti,ab. | 9809 |
| 18 |  | (pricing or funding or reimbursement).ti,ab. | 54204 |
| 19 | Hits of Rare disease/Orphan drugs | 1 or 2 or 3 | 41664 |
| 20 | Hits of HTA | 4 or 5 or 6 or 7 or 8 or 9 or 10 or 11 or 12 or 13 or 14 or 15 or 16 or 17 or 18 | 593486 |
| 21 | Hits of Rare disease/Orphan drugs and HTA | 19 and 20 | 909 |
| 22 |  | limit 21 to english language | 811 |
